# Pelvic Floor Muscle Strength and Bothersome Urinary Incontinence After Pregnancy: A Cohort Study

**DOI:** 10.1007/s00192-025-06085-2

**Published:** 2025-03-13

**Authors:** Sónia Cristóvão, Emelie Asplén, Josefin Borssén, Maria E. H. Larsson, Sabine Vesting

**Affiliations:** 1 Närhälsan Gibraltar Rehabilitation, Gothenburg, Sweden; 2 Department of Health and Rehabilitation, Unit of Physiotherapy, Institute of Neuroscience and Physiology, Sahlgrenska Academy, University of Gothenburg, Box 455, 40530 Gothenburg, Sweden; 3 Herrestad Midwifery Unit, Västra Götalandsregionen, Uddevalla, Sweden; 4 Institute of Health and Care Sciences, Sahlgrenska Academy, University of Gothenburg, Gothenburg, Sweden; 5 Gibraltargatan Midwifery Unit, Västra Götalandsregionen, Gothenburg, Sweden; 6 Research, Education, Development and Innovation, Primary Health Care, Region Västra Götaland, Gothenburg, Sweden; 7 Region Västra Götaland, Research, Education, Development and Innovation, Primary Health Care, Gothenburg, Sweden; 8 School of Rehabilitation Sciences, University of Ottawa, Ottawa, ON Canada; 9 Arvid Wallgrens Backe Hus 2, 41346 Gothenburg, Sweden

**Keywords:** Midwifery, Pelvic floor dysfunction, Perinatal health, Physiotherapy, Postpartum, Women’s health

## Abstract

**Introduction and Hypothesis:**

Postpartum urinary incontinence (UI) is prevalent, and women with bothersome UI tend to seek more help. This study was aimed at evaluating the association between pelvic floor muscle (PFM) strength and bothersome UI in the 1st year postpartum.

**Methods:**

A prospective cohort study was conducted with 504 participants. UI was evaluated by the International Consultation on Incontinence Questionnaire Short Form (online) and PFM strength was assessed via vaginal palpation (Modified Oxford Scale, MOS), at 3, 6, 9, and 12 months postpartum. Logistic regression analysis was used to analyze the data.

**Results:**

At 3 months postpartum, 52% of women had a PFM strength of ≥ 3 MOS, increasing to 84% at 12 months. 42% of women reported UI at 3 months, which remained unchanged by 12 months. PFM strength ≥ 3 MOS was moderately associated with less UI at 3 months (OR = 0.63, 95% CI 0.42–0.94) and at timepoints 6, 9, and 12 months. Antepartum UI was strongly associated with postpartum UI at all time points: 3 months (OR = 10.23, 95% CI 4.90–21.37), 6 months (OR = 7.75, 95% CI 3.95–15.21), 9 months (OR = 9.95, 95% CI 4.61–21.47), and 12 months (OR = 4.55, 95% CI 2.29–9.04). Grade 2 perineal tears were moderately associated with UI at 9 months postpartum (OR = 1.82, 95% CI 1.11–3.0).

**Conclusions:**

A stronger pelvic floor was associated with less bothersome UI in the 1st year postpartum. UI during pregnancy was most strongly associated with bothersome UI after childbirth. Antenatal screening for UI and promoting PFM training may be warranted to support postpartum recovery and minimize UI.

## Introduction

Pregnancy and childbirth are life events that involve significant physical and muscular changes, which can impact a woman’s quality of life and health. Childbirth is associated with altered pelvic floor muscle (PFM) function. Perineal tears and levator ani injury are risk factors for pelvic floor dysfunction such as urinary incontinence (UI) [1]. Approximately 30% of women who have given birth experience UI, and this prevalence remains unchanged up to 12 years postpartum [2]. Few women seek help for UI owing to shame or insufficient knowledge about pelvic floor dysfunction, or that help exists [3]. Women who do seek help experience their UI as more bothersome, having greater interference in their daily life [3]. Experiencing bothersome UI may lead women to restrict or avoid daily activities, exercise, and sexual activity owing to fear of leakage, which may contribute to lower quality of life, health, and functioning [4, 5].

Urinary continence is maintained through an interplay of neural and muscular controls [6]. Contraction of the PFMs results in a lift and compression of the urethra and inhibition of detrusor activity, which is important for preventing UI, particularly during increased intra-abdominal pressure [7]. Therefore, adequate PFM function is important to maintain continence. Moreover, a baseline assessment of PFM function is important to evaluate changes in muscle strength over time. Currently, however, there is no gold standard method for assessing and quantifying PFM strength.

Vaginal palpation using the Modified Oxford Scale (MOS) for assessing PFM strength is a commonly used and reliable method for postpartum PFM assessment among pelvic health physiotherapists in primary care, and has been proven to have moderate to strong correlation with technical assessment methods [8, 9]. Although several studies show an association between UI and PFM weakness, these studies often use technical devices [10], making it difficult to interpret reference values for PFM assessment by vaginal palpation. Additionally, there is no consensus on what is considered normal values for PFM strength [7] and there is a paucity of knowledge on the natural recovery of the PFM after childbirth [11].

The aim of this study is to address this gap by investigating the association between PFM strength, as assessed by vaginal palpation, and bothersome UI during the 1st year postpartum. Additionally, we aimed to identify associations between maternal and obstetrical variables and bothersome UI after childbirth.

## Materials and Methods

### Study Design and Settings 

For this article, data from a prospective observational cohort study “Afterbabybodystudy” (clinicaltrials.gov: NCT03703804) was utilized. The STROBE (Strengthening the Reporting of Observational Studies in Epidemiology) guidelines were used to report this study. Recruitment began in September 2018 and concluded in February 2020. Inclusion criteria were women over 18 years of age who understood Swedish and had given birth 8–12 weeks earlier (vaginal or cesarean delivery). Exclusion criteria included chronic pelvic and back pain, and major perineal tears (grade III or IV). Participants were recruited through maternal health and childcare clinics in Region Västra Götaland, Sweden, and via social media. Women who were interested in participating in the study initiated contact with the researcher (SV). Data were collected through clinical assessments and electronic questionnaires at four time points during the 1st year postpartum, specifically every 3 months from 3 to 12 months postpartum.

#### Clinical Assessment and Questionnaire

Strength of the PFMs was assessed through vaginal palpation, using a version of the Modified Oxford Scale (ordinal scale 0–5) tested for its reliability in a previous study [8]. Six physiotherapists at three rehabilitation centers in Region Västra Götaland conducted the assessments following a standardized protocol for assessment patterns, participant positioning, instructions, measurement points, and documentation. The physiotherapists had several years of experience in PFM assessment and were blinded to the participant’s symptoms and each other’s results.

Participants were informed about the study’s purpose and provided signed informed consent. To ensure comfort and understanding, they were educated on pelvic floor anatomy and function and shown how the vaginal palpation would be performed using a pelvic floor model. Assessments were discontinued in the case of severe discomfort or pain. Initially, participants performed a "test round" of vaginal muscle contractions without specific technique instructions. This was followed by three maximum force contractions, each held as long as possible, with 15-s pauses between attempts. The strongest contraction was rated using the MOS. To be rated MOS ≥ 3 on the 0–5 scale, the contraction needed to include both a squeezing and a lifting component, defined as a correct pelvic floor contraction [12]. No instructions or information to participate in pelvic floor training was provided to the participants before or after the assessments.

Before the clinical assessment, participants completed an electronic questionnaire measuring experienced UI using the Swedish version of the International Consultation on Incontinence Questionnaire-Urinary Incontinence Short Form (ICIQ-UI SF), which scores UI frequency, severity, and impact on everyday life on a scale of 0–21. As not all women experience UI as bothersome or something requiring treatment [13], only participants reporting that UI interfered with their daily life (rated as a score of ≥ 1 on ICIQ-UI SF question 3) were defined as having bothersome UI and included in the analysis. Bothersome UI is hereafter referred to as UI for the purpose of this article.

Covariates collected at 3 months postpartum included maternal age, BMI, antepartum UI (yes/no), parity (primi- or multipara), mode of most recent delivery (vaginal or cesarean), and self-reported second-degree perineal tear/episiotomy.

### Statistical Analysis

All data were analyzed using SPSS software version 25.0. For continuous data, the mean (M), standard deviation (SD), and range were reported. The data from participants who were not able to contract the PFMs and pushed instead were excluded from the analysis. PFM strength, originally rated on a scale from 0 to 5 using the MOS, was dichotomized into weak (< 3) and strong (≥ 3) categories owing to a high concentration of data in the middle of the scale.

To examine the association between UI and the independent variables, logistic regression analyses were conducted at four time points (3, 6, 9, and 12 months postpartum). Women without bothersome UI at 3 (*n* = 292), 6 (*n* = 334), 9 (*n* = 334), and 12 (*n* = 325) months postpartum were not included in the analyses. Multicollinearity tests confirmed no significant correlations between the variables. For this study, we interpreted odds ratio (OR) values as follows: OR 0.71–1 and 1.0–1.4 as a weak association, OR 0.41–0.7 and 1.5–3.0 as a moderate association, and OR 0–0.4 and > 3.0 as a strong association.

Regarding the variable "second-degree perineal tear/episiotomy," 63 women who had had cesarean deliveries did not provide responses. In the regression analysis, these participants were included in the "no rupture" group. Excluding them or counting them as "missing values" would have resulted in their exclusion from all variables, preventing the analysis from being performed. No tear and grade 1 perineal tear were classified as “no rupture.” Grade 2 perineal tear and episiotomy were classified as “second-degree perineal tear/episiotomy.” Regarding mode of delivery, vaginal delivery was 0 and cesarean section 1 in the analysis.

## Results

**Fig. 1:**
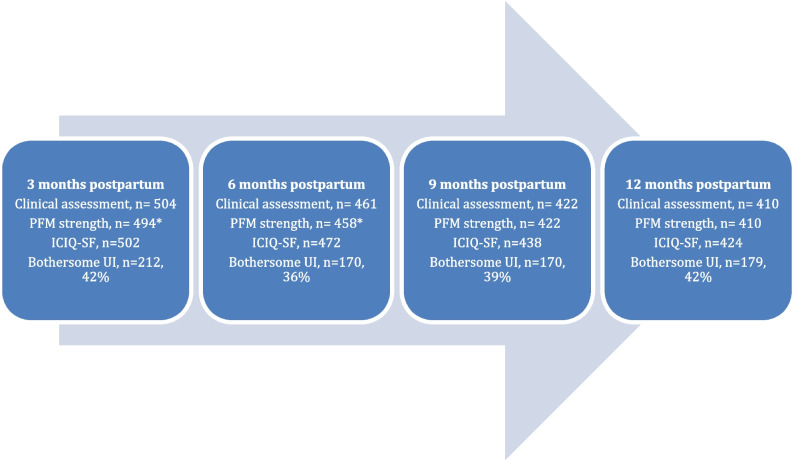
Flow chart of study participation and bothersome urinary incontinence (UI) during the 1st year postpartum. *Asterisk* indicates reduced number owing to incorrect pelvic floor contraction (straining). *PFM* pelvic floor muscle, *ICIQ-UI* Incontinence Questionnaire-Urinary Incontinence

A total of 504 participants were included in this study. Mean age was 33.1 years (SD 3.6, range 24–46) and mean BMI was 24.5 (SD 3.3, range 17–37). A total of 315 women were primipara (62.7%) and 187 were multipara (37.3%). The majority of participants (*n* = 438) had had a vaginal delivery, including 34 vacuum extractions, whereas 64 women (12.7%) had had a cesarean delivery (Table 1). A detailed description of the participants’ characteristics is reported in an earlier publication [4]. All 504 participants underwent a clinical assessment of PFM strength at 3 months postpartum, and 503 completed the questionnaire. Throughout the study, 18.7% of participants dropped out of the clinical assessment, and 15.9% dropped out of the questionnaire (Fig. 1).

**Table 1:** Characteristics of participants across the 1st year postpartum

Characteristics	Data
Age, mean (SD)	33.1 (3.6)
BMI, mean (SD)	24.5 (3.3)
Education, *n* (%)	
Primary school, high school, or adult education	46 (9.1)
University or college education < 3 years	46 (9.1)
University or college education ≥ 3 years	411 (81.5)
Parity, *n* (%)	
Primipara	315 (62.5)
Multipara	187 (37.1)
Mode of delivery, *n* (%)	
Vaginal delivery	438 (86.9)
Instrumental delivery	34 (6.7)
Cesarean section	64 (12.7)
Breastfeeding at 3 months, *n* (%)	
No	46 (9.1)
Part-time	45 (8.9)
Yes	412 (81.7)
Antenatal UI, *n* (%)	67 (13.3)
Bothersome UI, *n* (%)/ICIQ-UI score, mean (SD)	
At 3 months	212 (42.1)/6.2 (3.0)
At 6 months	172 (36.1)/5.9 (2.9)
At 9 months	170 (38.8)/6.0 (3.0)
At 12 months	179 (42.2)/5.8 (3.1)
Pelvic floor muscle strength ≥ 3, *n* (%)	
At 3 months	257 (51.9)
At 6 months	336 (72.9)
At 9 months	340 (80.6)
At 12 months	347 (84.4)

*BMI* Body Mass Index, *SD* standard deviation, *UI* urinary incontinence, *ICIQ-UI* Incontinence Questionnaire-Urinary Incontinence

**Fig. 2:**
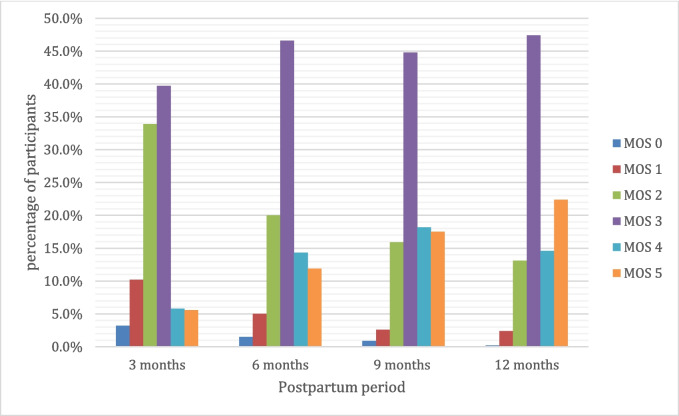
Distribution of pelvic floor muscle strength 3–12 months postpartum measured on the Modified Oxford Scale (MOS; 0–5)

At 3 months postpartum, 257 participants (52.0%) had a PFM strength of MOS ≥ 3. This number increased to 347 participants (84%) by 12 months postpartum (Fig. 2). A strong pelvic floor, indicated by a MOS score of ≥ 3, was moderately associated with reduced UI at 3, 6, and 12 months postpartum. The OR for reduced UI was 0.63 (95% CI 0.42–0.94) at 3 months and 0.41 (95% CI 0.23–0.76) at 12 months, indicating a decreasing likelihood of experiencing UI with stronger pelvic floor muscles over time (Table 2).

**Table 2:** Logistic regression analysis of risk factors for bothersome urinary incontinence

	3 months postpartum				6 months postpartum				9 months postpartum				12 months postpartum			
	Exp (B)	95% CI		*p*^2^	Exp (B)	95% CI		*p*^2^	Exp (B)	95% CI		*p*^2^	Exp (B)	95% CI		***p***^2^
		Lower	Upper			Lower	Upper			Lower	Upper			Lower	Upper	
Age	1.04	0.98	1.10	0.22	1.05	0.99	1.12	0.11	1.06	1.00	1.14	0.06	1.07	1.00	1.14	*< 0.01*
BMI	1.08	1.02	1.15	*0.01*	1.05	0.98	1.12	0.15	1.06	0.99	1.14	0.11	1.04	0.97	1.11	0.23
Parity	1.03	0.66	1.60	0.90	0.62	0.38	1.02	0.06	0.98	0.59	1.63	0.95	0.97	0.59	1.58	0.90
Birth weight ≥ 4000 g	0.81	0.49	1.36	0.43	1.12	0.65	1.90	0.69	0.86	0.48	1.52	0.59	0.67	0.38	1.20	0.18
Delivery mode^1^	0.45	0.23	0.90	*0.02*	0.70	0.35	1.40	0.32	0.41	0.18	0.91	*0.03*	0.65	0.32	1.30	0.22
Grade 2 perineal tear	1.17	0.75	1.83	0.48	1.26	0.78	2.01	0.35	1.82	1.11	3.00	*0.02*	1.30	0.79	2.11	0.30
Antenatal UI	10.23	4.90	21.37	*< 0.01*	7.75	3.95	15.21	*< 0.01*	9.95	4.61	21.47	*< 0.01*	4.55	2.29	9.04	*< 0.01*
PFM strength MOS ≥ 3	0.63	0.42	0.94	*0.02*	0.52	0.33	0.83	*0.01*	0.59	0.34	1.04	0.07	0.41	0.23	0.76	*< 0.01*
Constant	0.03			0.01	0.04			0.02	0.02			0.01	0.05			0.04

*BMI* Body Mass Index, *CI* confidence interval, *MOS* Modified Oxford Scale, *PFM* pelvic floor muscle

*Italic *p* value indicates statistical significance *p* ≤ 0.05

^a^Delivery mode: vaginal delivery = 0, cesarean section = 1

Antepartum UI had the strongest association with postpartum UI, with OR decreasing from 10.23 (95% CI 4.90–21.37) at 3 months to 4.55 (95% CI 2.29–9.04) at 12 months; demonstrating a diminishing but still significant influence over the 1st year postpartum. At 9 months postpartum, having a second-degree perineal tear or undergoing an episiotomy was moderately associated with UI, with an OR = 1.82 (95% CI 1.11–3.00; Table 2).

At 12 months postpartum, age was weakly associated with an increase in the risk of UI, with an OR = 1.07 (95% CI 1.00–1.14). At 3 months postpartum, higher body mass index (BMI) had a weak association with UI, with an OR = 1.08 (95% CI 1.02–1.15), and vaginal delivery had a moderate association with UI, with an OR = 0.45 (95% CI 0.23–0.90; Table 2).

## Discussion

The main findings of this study were that increased PFM strength, assessed by vaginal palpation, was moderately associated with experiencing less bothersome UI in the 1st year after childbirth. Antepartum UI was most strongly associated with postpartum UI. Vaginal delivery and second-degree perineal tears were moderately associated with UI at 3 and 9 months postpartum respectively. Finally, whereas PFM strength increases throughout the 1st year postpartum, the frequency of UI tends to decrease slightly during the first 6 months postpartum before increasing again from 9 to 12 months.

Strong PFMs, corresponding to MOS ≥ 3, was moderately associated with reduced UI at 3, 6, and 12 months postpartum, suggesting that PFM strength might be important for maintaining continence. Although Baracho et al. [14] reported that PFM strength was a strong predictor of stress UI at 5–7 months postpartum, two other studies did not find significant associations between PFM strength and UI at 6 or 12 months postpartum respectively [15, 16]. Neither of these studies evaluated bothersome UI and only included primigravida, which may explain the difference compared with our findings. Diez-Itza et al. [16] did, however, report that the mean value of PFM strength at 6 months postpartum was significantly lower among incontinent women. Other studies have also reported that continent women have stronger PFMs [17], supporting our finding that PFM strength is important for reduced postpartum UI.

At 9 months postpartum PFM strength was not significantly associated with UI. This may be explained by changes in lifestyle habits at this point in time [18]. Furthermore, PFM strength was assessed in supine position, where muscles are not maximally loaded. Therefore, an increase in activity levels at 9 months postpartum may load the PFMs to a degree that is greater than their functional capacity in supine position. The fact that this association is evident again at 12 months postpartum may be explained by the fact that women may adjust or reduce their activity levels when they begin to experience UI [4]. Additionally, the pelvic floor may adapt through the principles of specificity and progressive overload to be able to meet these demands over time [19]. Further research is needed to fully understand what is happening at 9 months postpartum.

More than half (52%) of the participants had a PFM strength corresponding to MOS ≥ 3 at 3 months postpartum, increasing to 73% at 6 months postpartum and 84% by 12 months. Thus, the greatest improvement in PFM strength was evident during the first 6 months postpartum but is an ongoing process throughout the 1st year after childbirth. This is in line with findings that significant recovery of the muscles, nerves, and connective tissue occurs within the first 6 months after childbirth, after which improvements continue but are not statistically significant [20]. Another study found that although PFM strength improved between 6 and 12 months postpartum, the values had not returned to mid-pregnancy levels [11]. These findings support our results that recovery of PFM function occurs throughout the whole of the 1st year after childbirth. Our study adds to the information on PFM recovery for multiparous women. However, as we did not collect data on whether the women had participated in a home PFM training program, it is difficult to know if this improvement is due to natural increases in strength or to training.

Frequency of UI after childbirth decreased slightly from 3 months (42.1%) to 6 months postpartum (36.1%), increasing again from 9 months (38.8%) to 12 months (42.2%) postpartum. Our findings are similar to those of a recent meta-analysis, where authors found an initial drop in prevalence of UI in the first 3 months postpartum followed by a subsequent increase from 3 to 12 months [21]. This may be attributed to an increase in physical activity from 6 to 9 months, coupled with the slower recovery of PFM strength after 6 months postpartum [20]. Women should be educated about the recovery process of PFM function and assisted in a progressive return to physical activity.

The finding that although PFM strength increases throughout the 1st year postpartum, the frequency of UI tends to decrease slightly during the first 6 months postpartum before increasing again, suggests that factors other than muscle function might also be important for maintaining continence. Factors such as gestational diabetes mellitus, smoking, and duration of breastfeeding, for example, have also been reported to be associated with postpartum UI [22]. Although PFM strength is one piece of the puzzle of reducing UI in the postpartum period, other individual risk factors must also be considered by health care professionals.

Our finding that antepartum UI was most strongly associated with postpartum UI the 1st year after childbirth has been reported for both primi- and multiparous women in previous studies [15, 22, 23]. This stresses the importance of doctors, midwives, and physiotherapists screening for this condition during pregnancy, and serves as a clinical predictor of postpartum UI. Early identification is a low-risk strategy that allows health care providers to educate women at risk for UI and identify patients who may benefit from pelvic floor physiotherapy. A Cochrane review demonstrated a significant effect of antenatal PFM training on reduced postpartum UI [24], suggesting that pregnancy might provide a window of opportunity to educate all women on the importance of PFM training for the prevention of UI and should be investigated in future studies.

Having a second-degree perineal tear or undergoing an episiotomy was moderately associated with UI at 9 months postpartum. A paucity of studies examining this relationship exist, making comparisons difficult. However, similar to our findings, an association between perineal tears or episiotomy and UI at 12 months postpartum has previously been shown in primiparous women [25]. Our study adds information that this association and risk exists for multiparous women as well. These results suggest that women who have a second-degree perineal tear or undergo episiotomy might require more support and individualized advice at 9 months, when they tend to increase their physical activity levels, to prevent UI.

Associations between UI and higher BMI, vaginal delivery, and higher maternal age have been found in previous studies [26]. Whereas vaginal delivery and maternal age are nonmodifiable factors, interventions to mitigate increased BMI may be feasible during and after pregnancy. Wesnes et al. [27] found an association between weight loss and lower prevalence of UI among women with incontinence during pregnancy. Therefore, strategies that support safe weight loss after childbirth may be clinically important to reduce the prevalence of UI in the 1st year postpartum.

### Strengths and Limitations

Strengths of our study include the large sample size and the inclusion of both primipara and multipara women, which can increase generalizability, as well as the prospective cohort study design with multiple time points, which adds to the reliability of the results. One limitation is that there is no clear or widely accepted definition of bothersome UI. The ICIQ-SF is a measurement instrument with good construct validity [28] and has been validated in the perinatal population [29]. However, we chose a cut-off of ≥ 1 on the scale for how much UI interferes with daily life based on discussions within our research group. When comparing our results with those of others, readers should be observant of whether different cut-off points were chosen. Moreover, the choice of cut-off points for PFM strength are both a strength and a limitation. With a lack of previous studies in this field, even this cut-off point was chosen based on clinical experience and discussions within the research group. It can, however, be used in clinical practice where vaginal palpation is a common assessment method. Another limitation of this study is that we investigated only voluntary maximal PFM contraction. Other functions, such as PFM endurance, reflex activation ("the knack"), and relaxation, could also be important to investigate in relation to UI. However, because only slight to moderate reliability for assessing these functions via vaginal palpation has been shown [8], they were not included in the analysis. Further studies on these functions, using more reliable assessment methods, could help to explore this question further. That second-degree perineal tears/episiotomy were self-reported increases the risk for recall bias as participants answered this question 3 months after childbirth and we cannot rule out that women reported incorrectly owing to a lack of clinical knowledge of perineal tear grades. Finally, recruiting through advertising increases the risk for selection bias. The recruited women were highly educated (81.5% university or college, 3 years or longer), which can affect the generalizability of our results.

### Future Implications

Future studies should explore whether early identification, prevention, and treatment of UI can reduce its prevalence during pregnancy and in the 1st year postpartum.

## Conclusion

This study demonstrated that PFM strength is a significant factor associated with bothersome UI during the 1st year postpartum. Women presenting with a weak pelvic floor corresponding to MOS ≤ 2 at their postpartum check-up should be referred to a physiotherapist for individualized PFM rehabilitation. Although PFM strength is critical for continence, other factors, such as perineal trauma (e.g., second-degree tears or episiotomy), BMI, age, and delivery mode, also contribute to UI risk and PFM training may not resolve every case of UI. These findings highlight the need for a comprehensive approach in postpartum care, with tailored interventions for individual risk factors. Finally, antepartum UI emerged as the strongest predictor of postpartum UI, underscoring the importance of early identification and intervention during pregnancy. Health care professionals should prioritize antenatal screening for UI, promote PFM training when assessed to be appropriate, and provide individualized postpartum support to optimize recovery and minimize UI.

## Data Availability

The data that support the findings of this study are available from the corresponding author, [SC], upon reasonable request.
